# Three prolonged outbreaks of metallo-β-lactamase-producing *Pseudomonas aeruginosa* in an Upper Austrian hospital, 2017–2023

**DOI:** 10.1128/spectrum.00740-24

**Published:** 2024-08-20

**Authors:** Adriana Cabal, Anna Hörtenhuber, Yarub Salaheddin, Anna Stöger, Burkhard Springer, Stefan Bletz, Alexander Mellmann, Patrick Hyden, Rainer Hartl, Johannes Weinberger, Rick Conzemius, Markus Hell, Beatriz Daza-Prieto, Kathrin Lippert, Georg Steindl, Sandra Köberl-Jelovcan, Werner Ruppitsch

**Affiliations:** 1 Division for Public Health, Institute for Medical Microbiology and Hygiene, Austrian Agency for Health and Food Safety, Vienna, Austria; 2 Institute of Pathology, Upper Austrian Health Holding GmbH, Pyhrn-Eisenwurzen Clinical Centre Kirchdorf Steyr, Steyr, Austria; 3 Division for Public Health, Institute for Medical Microbiology and Hygiene, Austrian Agency for Health and Food Safety, Graz, Austria; 4 Institute of Hygiene, University Hospital Muenster and University of Muenster, Muenster, Germany; 5 Department of Statistics and Analytical Epidemiology, Austrian Agency for Health and Food Safety, Vienna, Austria; 6 National Reference Center for Antimicrobial Resistance, Institute for Hygiene, Microbiology and Tropical Medicine, Ordensklinikum Linz Elisabethinen, Linz, Austria; 7 Johannes Kepler University Linz, Medical Faculty, Linz, Austria; 8 Ares Genetics GmbH, Vienna, Austria; 9 MEDILAB, Teaching Laboratory of the Paracelsus Medical University, Salzburg, Austria; 10 Institute for Hospital Hygiene and Microbiology (IKM), Graz, Austria; Duke University, Durham, North Carolina, USA

**Keywords:** *Pseudomonas aeruginosa*, metallo-β-lactamase, cgMLST, outbreak, Austria

## Abstract

In spring 2022, an increase in metallo-β-lactamase-producing *Pseudomonas aeruginosa* (MBL-Pa) infections was detected in a hospital in Upper Austria. To identify the source of infection and to stop further transmissions, an epidemiological outbreak investigation including whole-genome sequencing (WGS)-based typing was conducted. The final case definition included cases admitted to the hospital between 2020 and 2023 with an MBL-Pa in one of the three genomic clusters identified. In addition, the investigation was extended to include historical cases from 2017. Core genome multilocus sequence typing was performed to assess the genetic relatedness between the isolates. Fifty-four clinical *P. aeruginosa* isolates and eight *P. aeruginosa* isolates from the hospital environment were obtained. All but nine isolates grouped into one of three genomic clusters (ST235/*bla*_VIM-1_, ST111/*bla*_VIM-2_, or ST621/*bla*_IMP-13_), which were considered to be distinct, prolonged outbreaks involving 47 out of 52 cases. The most likely source of infection for cluster 1 (ST111/*bla*_VIM-2_) and cluster 2 (ST235/*bla*_VIM-1_) was sinks in the intensive care unit (ICU) washroom. Cluster 3 clone (ST621/*bla*_IMP-13_) could have originated in the urology ward in 2020 and then spread to the ICU years later. However, the nosocomial origin of this clone could not be proven. In March 2023, following the implementation of control measures (gowning, patient isolation, screening, and daily disinfection), no further MLB-Pa was detected, and the outbreaks were considered to be over. As ICUs play an important role in the transmission of *P. aeruginosa*, emphasis should be placed on genomic surveillance, infection prevention, and control in such wards.

**IMPORTANCE:**

The significance of our work lies in the successful resolution of three prolonged outbreaks of MBL-Pa infections in a hospital in Upper Austria. Through a comprehensive epidemiological investigation coupled with WGS-based typing of *P. aeruginosa* isolates, the study identified three distinct genomic clusters responsible for prolonged outbreaks involving 47 cases. The investigation pinpointed sinks in the ICU washroom as the likely source of infection for two of the clusters. The study demonstrates the effectiveness of control measures such as hand hygiene, gowning, patient isolation, screening, and disinfection in stopping further transmission and bringing the outbreaks to a close. This underscores the critical role of genomic surveillance and control measures, particularly in high-risk settings like ICUs, in reducing nosocomial transmission of MBL-Pa infections.

## INTRODUCTION

*Pseudomonas aeruginosa* is an opportunistic Gram-negative bacterium that can be found ubiquitously in various niches, such as water, soil, and vegetation (1). However, it is considered as one of the top opportunistic pathogens capable of causing nosocomial infections in clinical settings, including pneumonia, urinary tract infections, wound infections, and septicemia (2). The incidence of *P. aeruginosa* infections is higher among intensive care unit (ICU) patients, who are at higher risk of infection due to their immunocompromised status (3, 4). First-line drugs to treat these infections are β-lactams and quinolones, followed by colistin and aminoglycosides. Particularly in ICUs, the persistence and spread of *P. aeruginosa* due to biofilm formation in sinks and siphons used for patient care is a major problem (5). Many infections caused by *P. aeruginosa* are difficult to treat because of the acquisition of antibiotic resistance determinants, particularly to carbapenems (6, 7) in addition to the intrinsic resistance that this bacterium possesses (8). The emergence and spread of carbapenem-resistant *P. aeruginosa* strains are a major global public health problem requiring control of carbapenem resistance, surveillance, antimicrobial stewardship programs, and rapid and accurate diagnostic tools (9). As a result, the World Health Organization included *P. aeruginosa* on its 2017 priority list of critical pathogens for which new antibiotics are urgently needed (10).

Carbapenem resistance in *P. aeruginosa* is due to overexpression of efflux pumps and intrinsic AmpC β-lactamases, porin loss or alteration, and acquisition of carbapenemases (11). Porin loss or porin alteration reduces the permeability of the bacterial wall, as is the case with the porin OprD (12). Metallo-β-lactamases (MBLs) such as VIM, IMP, or NDM, also known as class B carbapenemases, can hydrolyze a broad spectrum of β-lactams. *P. aeruginosa* can also acquire genes encoding extended-spectrum β-lactamases and aminoglycoside enzymes as well, but also mutations in the DNA gyrase or topoisomerase IV conferring fluoroquinolone resistance are described (13).

Regarding the genetic diversity of *P. aeruginosa*, a phylogenetic analysis based on multilocus sequence typing (MLST) identified the high-risk sequence types (STs) ST111 and ST235 as globally distributed, persistent, non-clonal, and multidrug resistant, often causing hospital outbreaks (11). ST621 is an emerging clone commonly associated with IMP-13, which was first detected in Italy in 2001 (14, 15). *P. aeruginosa* has an extensive repertoire of mobile genetic elements in relation to AMR. Plasmids, class 1 integrons, transposons, or genomic islands are some of the most relevant ones (11). Regarding virulence, some specific STs, such as ST235, can express virulence factors such as ExoU, making them more pathogenic, especially in patients with pneumonia (7).

The most recent resistance report in Austria for the year 2021 (16) indicated that 12.4% of the *P. aeruginosa* isolates analyzed by the national reference laboratory were carbapenem resistant. In 2007, metallo-β-lactamase-producing *Pseudomonas aeruginosa* (MBL-Pa) isolates were reported for the first time in Austria (17). Three were obtained in an Upper Austrian hospital (hospital A) and had either *bla*_IMP-13_ or *bla*_VIM-2_ and were ST621 and ST111, respectively. Several years later, between December 2021 and March 2022, the same hospital observed an increase in infections caused by MBL-Pa, indicating a possible outbreak.

In order to identify the possible source of infection and to stop further transmissions, an epidemiological outbreak investigation including whole-genome sequencing (WGS)-based typing of *P. aeruginosa* isolates was initiated.

## RESULTS

### Outbreak description

Initially, 52 cases were identified as MBL-Pa at hospital A between 2017 and 2023, but only 49 of these matched the initial case definition, as 3 of them were historical patients from 2017. Overall, cases were between 34 and 86 years of age (interquartile range = 15); 41 were male; and 11 were female. Forty-one cases lived in Upper Austria, 10 in Lower Austria, and 1 in Carinthia (Fig. S1). All cases were found to be infected with *P. aeruginosa*, except case 21, who was only colonized.

The median time from hospital admission to isolation for MBL-Pa was 22 days (range 0–119 days). Among deceased cases, the median time from hospital admission to death was 82 days. The mortality rate was 28.85% (*n* = 15), but according to medical records, none of the cases died from MBL-Pa infection but from other comorbidities. Most cases were patients hospitalized for diseases or disorders such as cancer (28.85%), gastrointestinal illnesses (17.31%), or urinary tract conditions (13.46%). Thirty-seven cases were classified as nosocomially acquired and 15 cases as community acquired. The demographics of the 52 cases are shown in Table 1.

**TABLE 1:** Demographics of the 52 initial MBL-Pa cases from hospital A^*a*^

Patient	Sex	Age	Province of residence	Source of isolation	Hospitalization reason	Sampling ward	Probable acquisition source	Patient status
pt1	M	63	Upper Austria	Bronchial secretion	Respiratory disease	21A	Nosocomial	Deceased
pt2	M	75	Lower Austria	Midstream urine	Urinary tract disease	12A	Community	Discharged
pt3	M	75	Upper Austria	Skin and soft tissue	Skin and soft tissue disorder	13B	Community	Discharged
pt4	M	83	Upper Austria	Surgical site	Cardiovascular disease	Out sur	Nosocomial	Deceased
pt5	M	68	Lower Austria	Skin and soft tissue	Gastrointestinal disease	23	Nosocomial	Discharged
pt6	F	63	Upper Austria	Surgical site	Gastrointestinal and urological disease	23	Nosocomial	Discharged
pt7	M	62	Upper Austria	Midstream urine	Cancer	12A	Nosocomial	Discharged
pt8	M	61	Upper Austria	Catheter related	Cardiovascular disease	24	Nosocomial	Discharged
pt9	M	73	Lower Austria	Surgical site	Gastrointestinal disease	23	Nosocomial	Discharged
pt10	M	47	Upper Austria	Surgical site	Skin and soft tissue disorder	24	Nosocomial	Discharged
pt11	F	64	Upper Austria	Skin and soft tissue	cardiovascular disease	13A	Nosocomial	Discharged
				Rectal swab		24	Nosocomial	
pt12	M	67	Upper Austria	Catheter related	Gastrointestinal disease	21A	Nosocomial	Deceased
pt13	F	66	Upper Austria	Blood culture	Unknown	30A	Nosocomial	Deceased
pt14	M	64	Upper Austria	Catheter related	Urinary tract disease	12A	Nosocomial	Discharged
				Catheter related		12A	Nosocomial	
pt15	M	68	Upper Austria	Bronchial secretion	Gastrointestinal disease	21A	Nosocomial	Discharged
pt16	M	36	Carinthia	Catheter related	Unknown	13B	Nosocomial	Discharged
pt20	M	66	Upper Austria	Surgical site	Cardiovascular disease	24	Nosocomial	Deceased
pt21	M	75	Upper Austria	Rectal swab	Urinary tract disease	12A	Nosocomial	Discharged
pt22	M	82	Lower Austria	Catheter related	Cancer	13A	Nosocomial	Discharged
pt23	F	81	Upper Austria	Bronchial secretion	Skin and soft tissue disorder	21A	Nosocomial	Discharged
pt24	M	53	Upper Austria	Catheter related	Respiratory disease	21A	Nosocomial	Deceased
pt25	M	70	Upper Austria	Sputum	Cancer	61A	Nosocomial	Discharged
pt26	M	79	Upper Austria	Throat swab	Cancer	21B	Nosocomial	Deceased
pt27	M	80	Upper Austria	Catheter related	Cancer	53	Nosocomial	Deceased
pt28	F	56	Upper Austria	Surgical site	Cancer	Out sur	Nosocomial	Discharged
pt29	M	65	Lower Austria	Midstream urine	Urinary tract disease	12A	Community	Discharged
pt30	M	60	Upper Austria	Midstream urine	Urinary tract disease	12A	Community	Discharged
pt31	M	34	Upper Austria	Midstream urine	Cancer	12A	Community	Discharged
pt32	M	51	Lower Austria	Midstream urine	Urinary tract disease	12A	Community	Discharged
pt33	M	76	Lower Austria	Midstream urine	Genital disease	12A	Community	Discharged
pt34	M	43	Upper Austria	Midstream urine	Cancer	12A	Community	Discharged
pt35	M	61	Lower Austria	Midstream urine	Cancer	12A	Community	Discharged
pt36	M	55	Upper Austria	Throat swab	Gastrointestinal disease	24	Nosocomial	Deceased
pt37	M	67	Upper Austria	Catheter related	Cancer	12A	Nosocomial	Discharged
pt38	M	46	Upper Austria	Midstream urine	Cancer	12A	Community	Discharged
pt39	M	78	Upper Austria	Bronchial secretion	Cerebrovascular disease	60	Nosocomial	Discharged
pt40	M	84	Upper Austria	Catheter related	Gastrointestinal disease	30A	Nosocomial	Deceased
pt41	M	81	Upper Austria	Midstream urine	Cancer	12A	Community	Discharged
pt42	M	70	Upper Austria	Midstream urine	Cancer	12A	Community	Discharged
pt43	M	64	Lower Austria	Catheter related	Unknown	63	Community	Deceased
pt44	M	86	Upper Austria	Skin and soft tissue	Cancer	24	Nosocomial	Discharged
pt45	M	75	Upper Austria	Catheter related	Gastrointestinal disease	23	Nosocomial	Discharged
pt46	M	73	Upper Austria	Sputum	Gastrointestinal disease	60	Nosocomial	Deceased
pt47	F	50	Upper Austria	Catheter related	Urinary tract disease	53	Community	Deceased
pt48	F	82	Lower Austria	Catheter related	Cerebrovascular disease	51	Nosocomial	Discharged
pt49	F	74	Upper Austria	Bonchial secretion	Infectious disease	30A	Nosocomial	Deceased
pt50	M	76	Upper Austria	Midstream urine	Cancer	12A	Nosocomial	Discharged
pt51	F	65	Upper Austria	Midstream urine	Respiratory disease	62A	Community	Discharged
pt52	M	80	Upper Austria	Bronchial secretion	Unknown	21A	Nosocomial	Discharged
pt53	M	66	Upper Austria	Skin and soft tissue	Gastrointestinal disease	23	Nosocomial	Discharged
pt54	F	82	Upper Austria	Skin and soft tissue	Infectious disease	13A	Nosocomial	Discharged
pt55	F	61	Upper Austria	Skin and soft tissue	Trauma	21A	Nosocomial	Deceased

^*a*^ F, female; M, male; out_sur, outpatient surgery; pt, patient.

Since March 2023 and after the application of control measures, i.e., gowning care, enhanced hand hygiene, patient isolation, regular screening, and daily disinfection, no more MBL-Pas were detected, and the three outbreaks were considered to be over.

**Fig 1:**
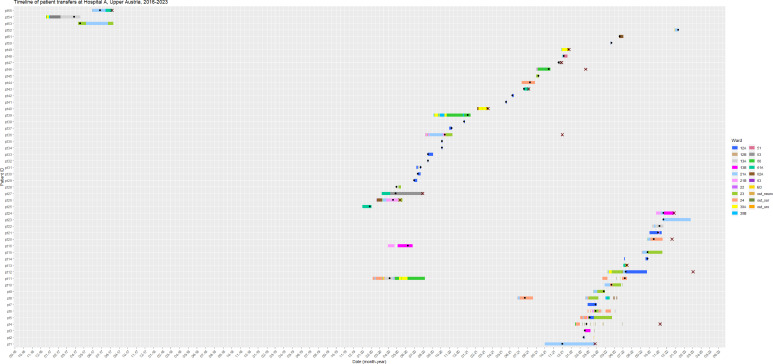
Timeline of the 52 patients’ stay in different wards at hospital A, 2016–2022. The black dots indicate the date of the first *P. aeruginosa* isolate, except for patient 11, who has two isolates collected at different times. Crosses indicate deceased patients.

Twenty-one cases were in an ICU ward (either 21A or 21B or both) at some point during their hospitalization. Of these, 19 had an epidemiological link to the ICU, meaning that all were in the ICU before or when the *P. aeruginosa* isolation took place. Figure 1 shows the movements for each patient between hospital wards.

A total of 54 isolates were obtained from the 52 cases. According to the source of isolation, most isolates were obtained from diverse catheter types (25.93%) or midstream urine (25.93%), followed by skin and soft tissue (12.96%) and surgical sites (11.11%). Patient (pt) 11 had already tested positive for MBL-Pa in 2020 and tested positive again in 2022. Two MBL-Pa isolates with different colony morphologies were obtained simultaneously from pt14. Three cases had *P. aeruginosa* isolates that were not MBL producers (pt25, pt39, and pt47), although they were resistant to meropenem and in total were resistant to at least four antibiotic classes.

### Phenotypic resistance

Phenotypic resistance data were obtained from hospital records. Using VITEK testing, we found that all 54 clinical isolates were resistant to piperacillin/tazobactam, ciprofloxacin, and meropenem and were susceptible to colistin. Ninety-eight percent were resistant to ceftazidime and cefepime, 96.3% to tobramycin, and 38.8% to amikacin. Only half of the isolates were tested for piperacillin, and all were resistant. Of the isolates tested for aztreonam (*n* = 41), 21.9% were resistant, while 78% showed intermediate resistance to this antibiotic. All isolates tested for imipenem (*n* = 41) were resistant. In three isolates, resistance to meropenem was detected, but no MBL was found. Fifty-three isolates were confirmed as multidrug-resistant *P. aeruginosa* (MDRPA), meaning that they were resistant to ≥1 antibiotic in ≥3 antibiotic classes. One of the non-MBL producers was extensively drug-resistant, meaning that it was resistant to ≥1 antibiotic in all but ≤2 antibiotic classes. All 19 isolates tested for ceftazidime/avibactam and ceftolozane/tazobactam were resistant to these antibiotics. All 18 isolates tested for meropenem/vaborbactam were resistant.

### Environmental investigations

Four bacterial isolates were obtained from the 2021 samples that tested positive for *P. aeruginosa* (*n* = 4) by matrix-assisted laser desorption ionization (MALDI) and PCR. Three isolates were confirmed as MBL-Pa by WGS (env1, env3, and env4) and one as *Stenotrophomonas maltophilia* (env2). All were obtained from the hygienic siphon in the ICU. Ten 2022 samples were PCR positive for *P. aeruginosa*, but no isolate could be obtained.

In 2023, 10 samples previously incubated in Schaedler’s broth were identified as *P. aeruginosa*. The samples were collected from sinks and basins, the patients’ bathroom, and the washroom. Five different MBL-Pa isolates were obtained from three samples (env5, env6, and env11). We also recovered *Pseudomonas stuzteri* (env12) from one sample and *Pseudomonas putida* (env10) from another sample.

### Sequencing results

**Fig 2:**
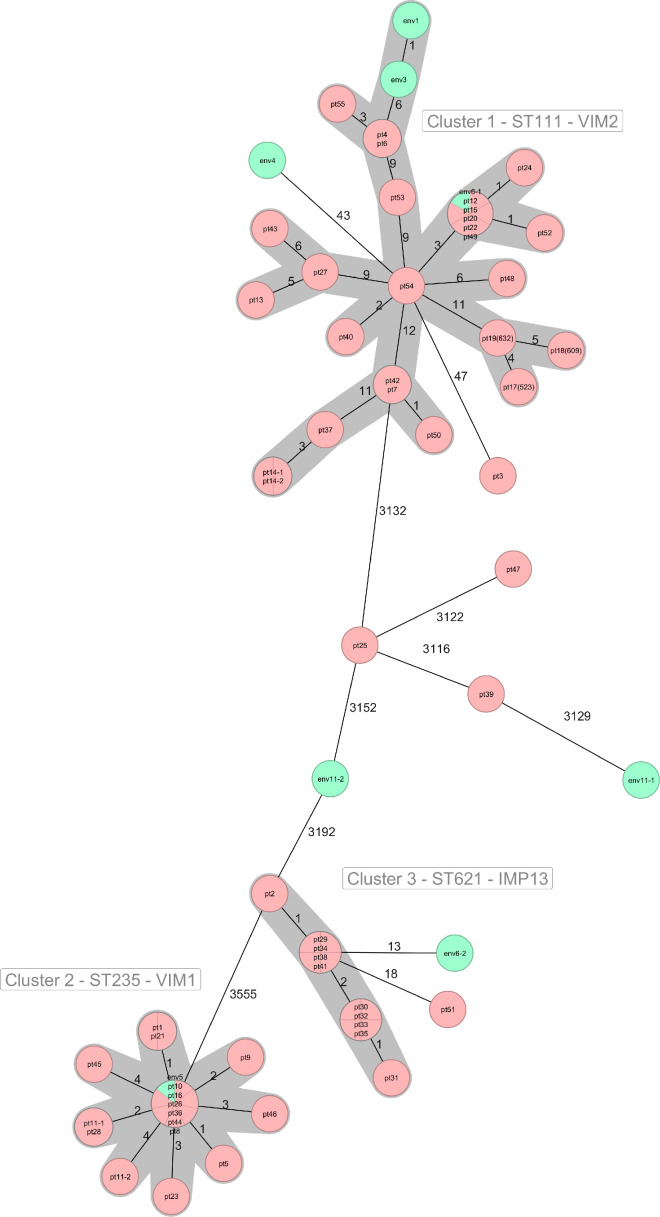
Minimum spanning tree of the 62 *P*. *aeruginosa* sequenced isolates from hospital A and other (*n* = 3) hospitals (hospitals B and C) based on their core genomes. References for each isolate are inside the circles. Pink isolates have a clinical origin. Green isolates have an environmental origin. The length of the lines between the circles does not correspond to the actual allele distances. The allele differences are indicated as numbers on the connecting lines. Clusters are highlighted in gray. The cluster threshold is 12 alleles.

We sequenced 62 *P*. *aeruginosa* strains from hospital A (54 clinical and 8 environmental). Using a cluster threshold of 12 alleles for core genome multilocus sequence typing (cgMLST) analysis, we detected 3 genomic clusters (clusters 1–3) and 9 singletons (Fig. 2). The final minimum spanning tree included three isolates already present in our database.

**Fig 3:**
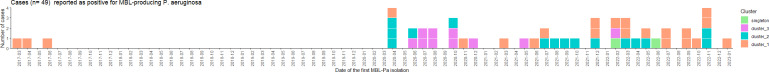
Epicurve of MBL-Pa confirmed cases (*n* = 49) from hospital A, 2017–2023. Cases in orange, blue, and pink are cases matching the final case definition (staying at hospital A from 2017 to 2023 with an MBL-Pa belonging to a genomic cluster). Cases with an MBL-Pa not belonging to any genomic cluster are “singletons” (green) and did not match the final case definition.

WGS confirmed the presence of an MBL gene in 51 isolates belonging to 49 of the 52 patients with a *P. aeruginosa* in hospital A. These 49 isolates included three patient isolates from 2017 that were part of cluster 1. The three remaining three cases were found to be infected with a non-MBL-producing *P. aeruginosa* and had isolates that did not belong to any genomic cluster. Two of the *P. aeruginosa* did not belong to any cluster and were considered as singletons. Thus, 47 cases were considered part of three different outbreaks according to their genomic clustering, and 44 cases matched the final case definition (Fig. 3; Fig. S2).

Cluster 1 consisted of 29 isolates of the high-risk clone ST111 - *bla*_VIM-2_. Twenty-three isolates were recovered from patients in hospital A, and three isolates belonged to patients in other hospitals (here referred to as hospitals B and C, located in Lower and Upper Austria, respectively). Three other isolates were recovered from the environment of hospital A. The patients with isolates in cluster 1 were in different wards, and nine of them died. All but two were classified as nosocomial cases. Eight had an epidemiological link to the ICU before or during the isolation of the MBL-Pa ST111. Four of these patients died. Isolates from three patients from hospitals B (523, pt17; 609, pt18) and C (632-pt19), which were already present in our database, belonged to cluster 1, and pt19 showed 11 allelic differences with isolate pt54, which was recovered in 2017, and thus represented one of the earliest isolates of this cluster. No epidemiological connection to the patients from hospital A was found. Two isolates (env1 and env3) from the hygiene samples of ward 21A differed from each other in only one allele, and one of them (env3) differed in six alleles from the closest patient isolates (pt4 and pt6). Patient 6 had an epidemiological link to ward 21A, having spent time in ward 21A/21B before the MBL-Pa was obtained. Within cluster 1, three subclusters (zero to one allelic difference) were identified. Subcluster A grouped patient isolates pt4 and pt6, who had spent time in ward 24, among other wards, before the MBL-Pa was isolated, and their corresponding wound isolates were identical. Subcluster B grouped patient isolates pt7, pt42, and pt50, which were obtained from midstream urine on ward 12A and differed from each other by zero to one allele. During their entire hospitalization, all three patients had been on this ward only, and all were cancer patients. Subcluster C comprised patient isolates pt12, pt15, pt20, pt22, pt24, pt49, and pt52 and the environmental isolate env6, which was collected at ward 21A. All isolates from this subcluster differed from each other by zero to one allele. The clinical isolates were obtained while the patients were in different wards and within a 27-month period (December 2021–March 2023). Six of the seven patients in this subcluster had spent time in the ICU (ward 21A) before or during the isolation of MBL-Pa.

Cluster 2 consisted of 17 isolates of the high-risk clone ST235, *bla*_VIM-1_, obtained between 2020 and 2022. It comprised 16 isolates from 15 patients, 3 of whom died. All cases within cluster 2 were classified as nosocomial. One isolate from our internal database from a patient treated at another hospital (pt16) in 2020 clustered with these isolates. This patient was found to have an epidemiological link to hospital A, having been previously hospitalized there and was then admitted to another hospital for surgery in the same year. An environmental isolate (env5) collected during sampling in ward 21A in 2023 showed zeroallelic differences from six of the patient isolates. Patients with isolates from cluster 2 stayed mainly on wards 21A or 21B, 23, and 24. Eleven patients with an epidemiological link to the ICU before or at the time of sampling had an MBL-Pa strain belonging to cluster 2. For those patients who had been in the ICU before or at the time of sampling, the odds of acquiring the cluster 2 clone (ST235) were approximately 5.25 times the odds of acquiring the cluster 1 clone (ST111) under the same conditions (*P* value = 0.0227). Cluster 2 isolates carried the *exo*U gene in addition to other virulence genes.

Cluster 3 consisted of 10 almost identical (zero to two allelic differences) ST621 IMP-13-producing MBL-Pas from the midstream urine of 10 male patients. All patients had been admitted to ward 12A at different times (2020 and 2022) and had been classified as community-acquired cases (isolation occurred <48 h after hospitalization).

Patient isolates pt25, pt39, and pt47 were singletons of other STs (132, 231, and 558) and did not harbor MBL genes.

Plasmid analysis indicated that the MBL genes were encoded on the chromosome. The gene encoding VIM-2 in the cluster 1 isolates was located on a chromosomal class 1 integron and was flanked by two aminoglycoside acetyltransferase *aacA29* genes. The gene encoding VIM-1 in cluster 2 isolates and *bla*_IMP-13_ in cluster 3 isolates was located on a chromosomal class 1 integron flanked by *aacA4-qacEdelta1-sul1*. Isolates in clusters 1 and 3 contained the transposon *tn4661*. Isolates in clusters 2 and 3 had the integrative conjugative element ICE6041(*tn4371*). Isolates in cluster 3 possessed the insertion sequence *IS6100*. In addition, *P. putida* was an IMP-13 producer, and the carbapenemase gene was flanked by *qacEdelta1-sul1* and *IS6100*, respectively. *S. maltophilia* and *Pseudomonas stutzeri* did not carry any acquired antibiotic resistance genes (ARGs).

**Fig 4:**
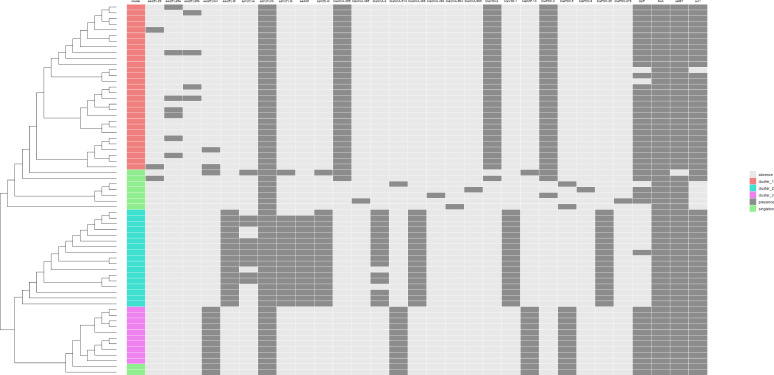
Heatmap representing the presence (dark gray) and absence (light gray) of ARGs for each of the 65 investigated *P. aeruginosa* isolates, by cgMLST cluster. On the left of the heatmap, a phylogeny is shown. Results are based on National Center for Biotechnology Information AMR FinderPlus. Only hits for >90% of length and >90% identity are shown.

Multiple alignment of the *oprD* gene sequences for all isolates and the PAO1 reference genome revealed several polymorphisms in this gene. Premature stop codons, deletions, and insertions were found in all cluster 1 and cluster 3 isolates. The *oprD* gene was either 1,325 or 1,326 bp. All isolates from the three clusters showed mutations, some of which resulted in amino acidic changes. Non-MBL producers showed different polymorphisms from MBL producers. One ST111 isolate harbored an *oprD* gene truncated by the insertion sequence ISPa26. *Amp*C changes were detected in all isolates (MBL-Pa and non-MBL-Pa) compared to the wild-type sequence of PAO1. These included polymorphisms in the codons R53Q (cluster 3), A71V (cluster 2), T79A (all clusters), V179L (cluster 2), and G365A (cluster 2). In addition, missense mutations, insertions, deletions, frameshift mutations, or premature stop codons were detected in *amp*R, *amp*D, PBP3 (*fts*L), PBP4 (*dac*B), *mex*R, and *mex*Z. In addition to MBL-encoding genes, all MBL-Pa isolates belonging to one of the clusters had intrinsic OXA-2- and/or OXA-50-like genes, *bla*PDC genes, and several genes conferring resistance to aminoglycosides, fosfomycin, fluoroquinolones, or sulfonamides. A more comprehensive summary of the ARG content detected with Illumina short-read sequencing for each isolate is presented in Fig. 4. In addition, genes mediating resistance against quaternary ammonium compounds were detected in all MBL-Pa isolates but not in the non-producers. All isolates in clusters 1 and 3 carried *qac*Edelta1, while all but three isolates in cluster 2 carried *qac*E.

Long-read sequencing confirmed a plasmid encoding *bla*_OXA-17_ in an ST235 clinical isolate and in an environmental ST621 singleton. The colistin resistance gene *basR* with an L71R mutation was identified in all six isolates sequenced with Nanopore.

## DISCUSSION

In this study, we used WGS to identify three distinct, prolonged outbreaks of MBL-Pa strains that occurred at hospital A from 2017 to 2023 and initially appeared as a single outbreak.

*P. aeruginosa* is a waterborne opportunistic pathogen with the ability to form biofilms, which can facilitate its long-term persistence. This makes it less susceptible to disinfectants and increase its risk of spread, particularly in hospital wards with immunocompromised patients (5), such as those with cancer. Almost 30% of the involved cases described here were indeed cancer patients. Regarding mortality, almost 30% of the patients in this study died. According to data from other studies, mortality in patients with an MDRPA is higher (45%) than that observed in those with a non-MDRPA strain (25%) (18). However, according to hospital records, the primary cause of death was not the *P. aeruginosa* infection but the primary disease. In addition, the median hospital stay of the patients who died was quite long (82 days). Indeed, prolonged hospital stay is considered a risk factor for acquiring MDRPA (19), especially in patients with a bloodstream infection, who are also at higher risk of mortality (20). Interestingly, only one case of bloodstream infection was identified in our study.

Three STs were identified here as outbreak clones: ST111, ST235, and ST621. ST111 has been implicated in both nosocomial and community-acquired infections, often exhibiting multidrug resistance and posing a significant clinical challenge. Outbreaks associated with ST111 may be related to contaminated medical devices or water sources within healthcare facilities (21, 22). The probable nosocomial transmission of the cluster 1 clone (ST111-*bla*_VIM-2_) in the ICU could not be prevented by the hygienic siphons present in this ward. One of the measures implemented to circumvent the problem of biofilms in hospitals, especially in ICUs, is the introduction of hygienic siphons that can disinfect themselves. Hygienic siphons clean and disinfect themselves, including the p-trap under the sink, thereby reducing the risk of biofilm formation (23). Previous studies have shown a significant reduction in the prevalence of Gram-negative bacteria, including *P. aeruginosa*, in hospital wards equipped with these types of hygienic siphons, although they do not eliminate them completely (23). Biofilms have the ability to regrow. Therefore, some experts have recommended water-free ICUs as the most effective solution to prevent the spread of such pathogens (24, 25). Two environmental isolates obtained from the hygienic siphon in 2021 differed by six and seven allelic differences, respectively, from the most closely related human isolates obtained in 2022, while the third environmental isolate obtained from the drainage sink in the ICU cleaning room in 2023 was identical to five other clinical isolates obtained in 2021 and 2022. This suggests that the drainage sink in the ICU washroom, followed by the hygienic siphon within the same ward, were the most likely sources of infection for isolates in cluster 1. Interestingly, an ST111-*bla*_VIM-2_ strain had already been detected in hospital A in 2007, but its sampling origin was unknown (17). Since WGS was not available at that time and the isolate was not preserved, it cannot be proven that it was related to those obtained in 2020–2022. In any case, it is important to acknowledge the potential existence of other unidentified sources of infection. Notably, cluster 1 isolates exhibited lower clonality, and this dissimilarity raises questions about the true relatedness of these isolates and the pathogen transmission. Although three isolates from hospitals B and C are grouped in this cluster, there was a high number of allelic differences (≥11 alleles) between them and the isolates from hospital A. This observation aligns with the absence of an epidemiological link between the isolates from both hospitals and those from hospital A. Therefore, we believe that setting a cluster threshold of 12 alleles to confirm transmission and identify the infection source for MBL-Pa may be too high. Consequently, we outlined potential subclusters within cluster 1 by lowering the cluster threshold. Pelegrin and colleagues already proposed a threshold of five or fewer cgSNPs for direct transmission (26). Given that both cgSNP and cgMLST analyses yield similar results (27), it is reasonable to conclude that isolates differing by ≤5 alleles can be considered closely related, suggesting potential transmission.

ST235 was identified in all isolates of cluster 2 and was associated with a likely nosocomial origin. Like ST111, ST235 has been responsible for numerous nosocomial outbreaks in healthcare settings worldwide, associated not only with multidrug resistance but also with increased virulence. Interestingly, all ST235 isolates carried *bla*_VIM-1_, which is rarely seen in this clone (28, 29), along with the *exoU* virulence gene, which is associated with poor outcomes in immunocompromised and pneumonia patients (30). The drainage basin of the ICU cleaning room was the only location where we found ST235-*bla*_VIM-1_ *P. aeruginosa* strains in environmental samples. Among all sequenced isolates, those from cluster 2 exhibited the highest degree of genetic similarity. An isolate obtained from the sink of the ICU cleaning room was entirely identical to the clinical isolates, despite the extended time span (2020–2023). This sink was the most likely source of infection for cluster 2 isolates. Moreover, patients carrying ST235 had an epidemiological link to the ICU, and this relationship was found to be significant.

All cluster 3 isolates belonged to ST621, which not only is a less common sequence type involved in outbreaks but also includes strains with transferable carbapenem resistance determinants (31). ST621-IMP-13-producing *P. aeruginosa* has been associated with urinary tract infections worldwide (32, 33), and all cases involved in our cluster 3 outbreak were positive for this pathogen in their midstream urine. While community-acquired outbreaks of *P. aeruginosa* are relatively rare compared to nosocomial ones, the presence of the cluster 3 clone (ST621-IMP-13), as indicated by the isolation dates, suggests a possible community origin. However, it remains challenging to definitively exclude a nosocomial origin. In 2023, we obtained an environmental MBL-Pa isolate, characterized as ST621-*bla*_IMP-13_, from the drainage sink of the ICU cleaning room, the same basin as for cluster 2 environmental isolates. This isolate exhibited a difference of 13 alleles compared to the cluster 3 clinical isolates. Although the environmental isolate exceeded the cluster threshold of 12 alleles, the observed time lag between the clinical isolates (2020–2021) and the environmental isolate (2023) may indicate evolution of this *P. aeruginosa* clone. This supports the theory that the cluster 3 clone originated in ward 12A in 2020 and then spread to ward 21A. According to the literature, ST621-*bla*_IMP-13_ *P. aeruginosa* was first identified in hospital A in 2007 (17). However, the isolate was not preserved and therefore not available for genomic comparison, making it impossible to prove the existence of the cluster 3 clone prior to 2020.

Phenotypic resistance to aminoglycosides, β-lactams, carbapenems, cephalosporins, monobactams, and fluoroquinolones was detected, as well as resistance to combinations of a β-lactam (BA) antibiotic with a β-lactamase inhibitor (BLI). Ceftolozane/tazobactam, ceftazidime/avibactam, and meropenem/vaborbactam are specifically formulated to enhance the effectiveness of β-lactam antibiotics against multidrug-resistant (MDR) Gram-negative bacteria, including *Pseudomonas aeruginosa*. However, MBLs such as VIM-1, VIM-2, and IMP-13 confer resistance to even these advanced BL/BLI combinations, therefore explaining the observed phenotypic resistance in our isolates (34).

However, OprD loss, elevated AmpC expression, and other chromosomal mutations in efflux pumps are considered to be the main mechanisms of resistance for these BA/BLI combinations (35, 36). Indeed, we detected the presence of polymorphisms in the *oprD* gene previously associated with reduced susceptibility to carbapenems (37, 38). Similarly, truncation of the *oprD* gene by *ISPa26* has been reported elsewhere (39). In addition, we found polymorphisms in *amp*C previously described in the literature for specific STs (40).

Of note is the detection of the L71R mutation in *basR*, which has been associated with colistin resistance (41). However, in our study, no phenotypic resistance to colistin was detected in any of the isolates carrying this mutation.

In addition to MBL-Pa, we identified a *P. putida* strain carrying IMP-13. Other species of the genus *Pseudomonas*, often regarded as environmental, have been associated with nosocomial cases or even hospital outbreaks of resistant clones, *P. putida* being one of them (42, 43). Moreover, this species has been suggested as an environmental reservoir of carbapenemases for *P. aeruginosa* (44).

Our study is not without its limitations. First, it is noteworthy that all cases except one were found to be infected. However, we cannot rule out the possibility of transmission occurring between unrecognized asymptomatic colonized patients. Second, it is important to recognize that our study lacks isolates from before 2017. This lack of historical data makes it difficult to definitively determine how long these three clones have been present in the hospital environment, as genetically similar strains may be isolated years apart due to the presence of biofilms. Finally, the number of isolates obtained from the environmental samples in 2021 and 2023 was relatively limited, and the origin of these isolates cannot be definitively determined. This limitation could be overcome by increasing the number of environmental samples to be collected as soon as new cases of MBL-Pa infection are detected. Nevertheless, our study provides valuable insights into potential sources of MBL-Pa infections. The presence of distinct genetic clusters and the observed epidemiological links allowed us to classify the isolates into three different prolonged outbreaks based on their genetic relatedness.

Since ICUs play an important role in the transmission of MBL-Pa, emphasis should be placed on infection prevention and control in such wards to avoid having a persistent environmental source of MBL-Pa. The detection of MBL-Pa closely related to clinical isolates in the hospital environment underscores the critical importance of ongoing genomic surveillance for *P. aeruginosa* and other Gram-negative bacteria.

## MATERIALS AND METHODS

### Hospital setting

Hospital A is located in Upper Austria and, together with another hospital, is part of the largest medical center in the region, with 620 beds. Hospital A alone receives 28,506 inpatients and 114,971 outpatients per year and consists of 11 different buildings.

To identify asymptomatic and symptomatic carriers and prevent transmission of MBL-Pa, the hospital conducts regular screening of at-risk patients (e.g., ICU patients) at admission, for those patients with a history of colonization or infection with any type of MDR bacterium, and for patients who have previously received carbapenem therapy. Screening includes stool or rectal swabs, throat or bronchial swabs, and wound swabs. In addition, the ICU is equipped with seven hygienic siphons whose traps are self-cleaning and self-disinfecting with heat and ultrasounds at regular intervals to prevent biofilm formation.

### Cases

The initial case definition included a colonized or an infected patient with a laboratory-confirmed MBL-Pa staying at hospital A between January 2020 (detection of the first case) and the end of the outbreak in January 2023. Patients were considered infected if there was tissue invasion from normally sterile sites (e.g., blood) or if they exhibited clinical signs corresponding to the swabbed sites (e.g., sore throat with signs of infection in the throat for throat swabs or gastrointestinal symptoms for rectal swabs).

Patients with a laboratory-confirmed MBL-Pa collected as far back as 2017 were also included for comparative analysis and to identify potential associations between past and current cases. The final case definition after obtaining the WGS results included a colonized or an infected patient with a WGS-confirmed MBL-Pa belonging to one of the three identified genomic clusters staying at hospital A between January 2020 and the end of the outbreak in January 2023.

For each patient, epidemiological data including sex, age, day of admission, day of discharge, history of ward/hospital transfer, day of first isolation of MBL-Pa, body site of collection, and patient outcome were obtained from medical records. All patients were followed up until discharge or death. The day of isolation was considered to be the day the first isolation of an MBL-Pa strain occurred. Subsequent dates of specimen collection (either screening or due to *P. aeruginosa* infection) with a negative or positive MBL-Pa test result were also recorded.

If the first isolate was obtained within 48 h of the patient’s admission to the hospital, the case was considered community acquired. If the first isolate was obtained more than 48 h after admission, the case was considered nosocomial. If only the screening sample of a given patient was positive, colonization rather than infection was assumed.

### Bacterial identification and susceptibility testing of clinical isolates

Clinical MBL-Pa isolates were collected by the hospital between 2020 and 2023. The species *P. aeruginosa* was identified by MALDI-time of flight mass spectrometry (MALDI Microflex; Bruker Daltonik, Bremen, Germany) after plating the sample on MacConkey agar (BD, Heidelberg, Germany).

For each patient isolate, antimicrobial resistance data were also retrieved from hospital records. Briefly, antibiotic susceptibility testing was performed by disk diffusion (Oxoid; Thermo Scientific, UK) for piperacillin/tazobactam, ciprofloxacin, ceftazidime, cefepime, meropenem, tobramycin, and amikacin. Cefiderocol, meropenem/vaborbactam and ceftolozane/tazobactam, and ceftazidime/avibactam were tested only when multidrug resistance was confirmed. The VITEK2 system from BioMerieux was used to confirm the disk diffusion results and to obtain the minimum inhibitory concentrations (MICs). VITEK2 was used to determine MIC values for colistin, and MICRONAUT MIC-Strip Colistin, Bruker Daltonic, was used after November 2021. For cefiderocol, meropenem/vaborbactam, and ceftolozane/tazobactam, no MIC values were available from the hospital records, but qualitative interpretations of antibiotic susceptibility were used instead. Later, these antibiotics were also routinely tested to determine their MICs.

Two different methods were used by the hospital’s microbiological laboratory to detect MBL-Pa. First, a PCR-based screening assay was performed using Eazyplex SuperBug complete C (AmplexDiagnostics GmbH, Gars-Bahnhof, Germany). Second, a modified Hodge test for ertapenem, imipenem, and meropenem was performed using the American Type Culture Collection 700603 *Klebsiella pneumoniae* strain. Ambiguous results were further confirmed by the reference laboratory. Results were interpreted according to the European Committee on Antimicrobial Susceptibility Testing (EUCAST) guidelines (45).

### Environmental sampling

Following the detection of MBL-Pa in the ICU in 2021, an environmental sampling was conducted in ward 21A in building 2. Water samples were taken quarterly in 2021 and 2022, and the siphons were sampled. A total of 100 samples were collected from the hygienic siphon, unclean work areas, sink drains, sink elbows, washer decontaminators, patients toilets, and showers. In 2023, approximately 120 samples were collected in ward 12A (urology ward or outpatient clinic) and the ICU ward (21A and 21B) from the water and the environment of doctors’ rooms, staff lounges, keyboards, bedpan washers, monitors, work carts, chairs, electrocardiogram equipment, showers, wash hand basins, siphons, disinfectant buckets, cleaning carts, equipment room, emergency bronchoscope, ultrasound device, door handles, and pneumatic tube system. All samples were collected using RODAC plates (Becton Dickinson, Heidelberg, Germany) and swabs (eSwab; Copan, Brescia, Italy). For 2023 samples, the swabs were also used for the PCR GeneXpert PA assay (Cepheid, Sunnyvale, CA, USA). Samples were cultured on Columbia blood agar, MacConkey agar, and Schaedler broth (Becton Dickinson) at 37°C for 24 h. *P. aeruginosa* was identified by MALDI (Microflex, Bruker Daltonik).

### Control and preventive measures

A gowning protocol for staff was enforced, and positive patients were isolated whenever possible. Basic hygiene measures were diligently maintained, including thorough hand hygiene with alcohol-based hand rubs, regular patient screening, and daily disinfection of the patient environment according to the hospital handbook on hygiene measures for MDR Gram-negative bacteria. After patient transfers, the entire bed area was thoroughly disinfected, and any items near the patient that could not be thermally or chemically disinfected were properly disposed of. When an increase in MBL-Pa was detected, user observations were initiated, accompanied by hygiene inspections and regular meetings involving all responsible personnel. In case of positive samples in the siphons, they were decalcified and disinfected with TPH protect at 0.5%, a solution based on quaternary ammonium compounds, and Perform at 1% (Schülke & Mayr, Norderstedt, Germany), an oxidizing agent. Regular sinks involved in the outbreaks were immediately replaced with new ones.

### WGS and genomic comparisons

Clinical and environmental *P. aeruginosa* isolates from 2020 to 2023 were sent on ESwabs (Copan) to AGES for Illumina WGS and subsequent typing. In addition, hospital A sent three historical MBL-Pa isolates in 2017 patients to be linked to the suspected outbreak cases.

Upon arrival at the laboratory, the isolates were plated directly onto Columbia blood agar (Becton Dickinson). After incubation for 24 h at 37°C, genomic DNA was isolated using the MagAttract HMW kit (Qiagen, Hilden, Germany). Library preparation was performed with Nextera XT (Illumina, San Diego, CA, USA). Paired-end sequencing was performed on a NextSeq2000 instrument (Illumina) with a read length of 2 × 150 bp, aiming for a minimum coverage of 30-fold. Raw reads were trimmed and *de novo* assembled using SPAdes v.3.11.1 (46). Contigs were filtered for a minimum coverage of 5× and a minimum length of 200 bp using Ridom SeqSphere^+^ software v.9.0.3 (47). Isolates were subtyped using classical MLST and cgMLST using SeqSphere^+^ v.9.0.3 (48). The resistome and virulence genes of all isolates were retrieved from the genomes using National Center for Biotechnology Information AMRFinderPlus (49) and VFDB (50). Point mutations in a selection of chromosomal genes associated with carbapenem resistance [*opr*D, *amp*R, *amp*D, PBP3(*fts*L), PBP4(*dac*B), *mex*R, and *mex*Z] were identified using BLAST v.2.2.12 (51). Mobile genetic elements including plasmids were detected with MOBSuite (52). A minimum spanning tree was generated using the cgMLST data, and the proposed cluster threshold of 12 alleles was applied. Furthermore, an in-house cgMLST database was searched for *P. aeruginosa* isolates from other hospitals (hospitals B and C) that clustered with hospital A isolates.

Long-read sequencing (Oxford Nanopore and PacBio sequencing) was performed on six and five randomly selected isolates to determine the location (chromosomal or plasmid) of the MBL genes in each strain. Briefly, libraries were prepared using the native barcoding ligation kit (SQK-NBD114.24; Oxford Nanopore Technologies, Oxford, UK). Sequencing was performed with a FLO-MIN114 R10.4.1 SpotON flow cell on a GridION (Oxford Nanopore Technologies) device for 72 h. SUP basecalling was executed with Guppy v.6.3.8, and reads were filtered using Filtlong v.0.2.1. Assembly was done using Flye v.2.9.2-b1786 (53) and was polished with Medaka (https://github.com/nanoporetech/medaka) using medaka_consensus v.1.8.0 (model r1041_e82_260bps_sup_g632). For WGS with PacBio, isolates were sent in ESwabs (Copan) to the Institute of Hygiene, Münster, Germany. Genomic DNA was isolated using the NEB Monarch Genomic Purification Kit (New England Biolabs, Ipswich, Massachusetts, USA). Sequence libraries were constructed using the SMRTbell Express Template Prep Kit v.2.0 according to the manufacturer’s recommendations (Pacific Biosciences Inc., Menlo Park, CA, USA). After the 15-h sequencing run on the Sequel IIe system, the resulting long reads were assembled using the “Microbial Assembly” pipeline with default parameters within the SMRT Link software v.11 (Pacific Biosciences Inc.).

The sequencing data generated in this study have been deposited in the Sequence Read Archive under BioProject accession number PRJNA1054342. Accession numbers for the sequences generated in this study can be found in Tables S1 and S2.

### Statistical analysis

We identified the number of patients who were in the ICU before or at the time of MBL-Pa isolation. We used a binomial logistic regression model to calculate whether being in the ICU before or during the isolation was associated with a higher likelihood of acquiring a particular clone.

## SUPPLEMENTAL MATERIAL

Figure S1 spectrum.00740-24-s0001.svg Map of Austria showing the distribution of the initial MBL-Pa cases.

Figure S2 spectrum.00740-24-s0002.svg Workflow diagram for inclusion and exclusion of cases and clinical isolates in the course of the outbreak investigation.

Table S1 spectrum.00740-24-s0003.xlsx Characteristics of the *P. aeruginosa* isolates sequenced in this study.

Table S2 spectrum.00740-24-s0004.xlsx Characteristics of the non-*P. aeruginosa* isolates sequenced in this study.

ASM does not own the copyrights to Supplemental Material that may be linked to, or accessed through, an article. The authors have granted ASM a non-exclusive, world-wide license to publish the Supplemental Material files. Please contact the corresponding author directly for reuse.
